# Implantation dermoid

**DOI:** 10.11604/pamj.2021.40.99.31816

**Published:** 2021-10-13

**Authors:** Surya Besant Natarajan, Krishna Prasanth Baalann

**Affiliations:** 1 Department of Community Medicine, Sree Balaji Medical College and Hospital, Bharath Institute of Higher Education and Research, Chennai, India

**Keywords:** Swelling, cyst, dermoid

## Image in medicine

True epidermoid inclusion cysts result from the implantation of epidermal elements in the dermis, and can result from any trauma or surgical procedure. Theoretically, any skin puncture, laceration or incision has the potential to implant epidermal elements into the dermis and subcutaneous tissue, where continued production of keratin and other epidermal products produces a thick-walled cyst containing white cheesy material. They are frequently seen in who are subject to repeated minor traumas to the hand. Human papillomavirus (HPV) infection, ultraviolet exposure and ecrine duct obstruction are also suggested to be the additional factors in the development of palmoplantar epidermoid cysts. A 57-year-old male patient, who is a farmer by occupation, presented with a swelling in the left palm for last 4 years. He had a history of thorn prick in his palm 5 years back. He noticed a swelling in his palm four and a half years back, which was about 0.5 cm in size at the onset. The swelling is increasing slowly in size to attain the present size of about 4 cm. No heterotopic ossification (H/O) pain, fever, discharge. On examination a swelling in the middle of the left palm, globular 4 cm in diameter, smooth surface, no redness over the swelling. The surgical treatment is needed for symptomatic cysts causing interference with function. The treatment requires careful complete excision, removal of the entire cyst wall is necessary to avoid the recurrence.

**Figure 1:**
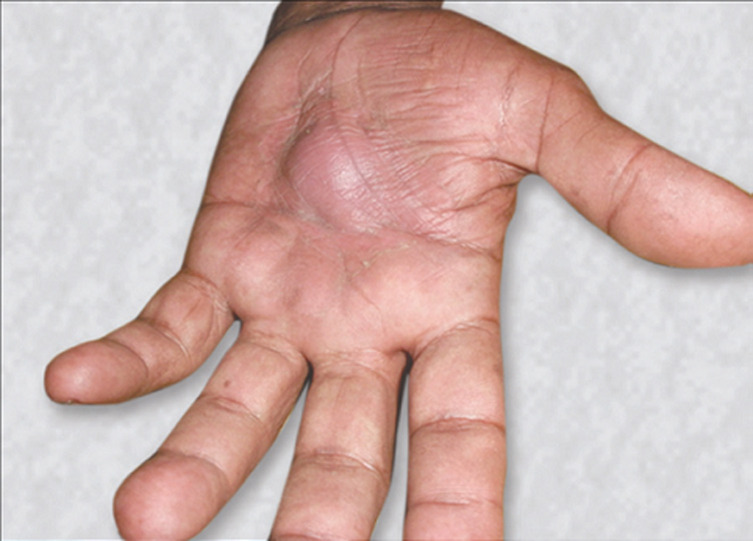
cystic swelling in the palm

